# Breast size, bra fit and thoracic pain in young women: a correlational study

**DOI:** 10.1186/1746-1340-16-1

**Published:** 2008-03-13

**Authors:** Katherine Wood, Melainie Cameron, Kylie Fitzgerald

**Affiliations:** 1 School of Health Science, Victoria University, Melbourne, Australia; 2 School of Human Movement, Recreation and Performance, Victoria University, Melbourne, Australia; 3 Centre for Ageing, Rehabilitation, Exercise and Sport, Victoria University, Melbourne, Australia

## Abstract

**Introduction:**

A single sample study was undertaken to determine the strength and direction of correlations between: a) breast size and thoracic spine or posterior chest wall pain; b) bra fit and thoracic spine or posterior chest wall pain and; c) breast size and bra fit, in thirty nulliparous women (18–26 years), with thoracic spine or posterior chest wall pain, who wore bras during daytime.

**Measures:**

Pain (Short Form McGill Pain Questionnaire), bra size (Triumph International), bra fit (Triumph International).

**Results:**

Most (80%) women wore incorrectly sized bras: 70% wore bras that were too small, 10% wore bras that were too large. Breast size was negatively correlated with both bra size (r = -0.78) and bra fit (r = -0.50). These results together indicate that large breasted women were particularly likely to be wearing incorrectly sized and fitted bras. Negligible relationships were found between pain and bra fit, and breast size and pain. Menstrual cycle stage was moderately positively correlated with bra fit (r = 0.32).

**Conclusion:**

In young, nulliparous women, thoracic pain appears unrelated to breast size. Bra fit is moderately related to stage of menstrual cycle suggesting that this research may be somewhat confounded by hormonal changes or reproductive stage. Further research is needed to clarify whether there is a relationship between breast size or bra fit and thoracic pain in women during times of hormonal change.

## Introduction

Back pain, including thoracic spinal pain, is a common, potentially disabling, routine presenting complaint to general practitioners [1]. Macromastia is the state of having disproportionately large breasts. Some macromastic women report breast pain and other symptoms, and the intuitively logical assumption is that breast size is the key influence on clinical presentation [2]. Clinical symptoms attributed to macromastia include neck, thoracic spine and shoulder pain, breast pain, headaches, grooving and associated pain caused by bra straps, intertrigo (inflammation of skinfolds), and ulnar nerve paresthesia [3].

Breast size and mass changes across the life-span [4,5] suggesting that macromastic symptoms may occur episodically during particular stages of life. Although these symptoms are widely reported, the relationship between breast size and symptoms is somewhat unclear. Breast mass and breast density appear to be important variables. Most outcome studies of reduction mammaplasties support the view that larger breasts equate to greater health burden [6-12] and demonstrate this relationship through symptom improvement post-surgery, but a recent review of 59 women who underwent reductions involving the removal of less than 1000 g of breast tissue showed that small reductions in breast mass may result in statistically significant improvements in macromastic symptoms [2].

Breast-related thoracic spinal pain is thought to result from changes in centre of gravity [12]. Findikcioglu and colleagues demonstrated that static spinal posture differs significantly according to breast size [13]. Letterman and Scheuter [12] argued that large breasts can increase cervical lordosis and thoracic kyphosis, shift the centre of gravity away from the spine and increase muscular effort required to maintain balance. They also suggested that large or heavy breasts may also lead to continuous tension on the middle and lower fibres of the trapezius muscle and associated muscle groups [12].

Greenbaum, Heslop and Morris [11] estimated that 70% of women wear bras that are incorrect sizes or poorly fitted. Ryan [14] proposed that elevation of the breasts in a bra increased downward forces on the outer scapula. He suggested that the posterior straps of a bra act as pulleys over the shoulders, effectively doubling the total downward pull on both shoulders. Associated neck, shoulder and back pain could then, at least partially, be attributed to fatigue in muscles that reverse scapular depression (eg: trapezius, serratus anterior). Bra-strap pressure is only somewhat linked to bust mass: small busted women with tight straps may experience considerable downward pressure on their shoulders [11,14].

Breast size and mass vary throughout life, influenced by hormonal changes, body fat composition, stage of reproductive cycle, and breast pathology [4,5]. Bra size, when fitted according to defined industry standards [15], may be used as an estimate of breast size. Across the lifespan and across the population, bra size is not a consistent measure of breast mass which is most accurately estimated from radiographic measures of volumetric density [16], but among healthy women who have never been pregnant or experienced breast pathology, bra size is likely to be a consistent measure [13]. In this study we examined the correlations between actual bra size (as an estimate of breast size), bra fit, and point-in-time reporting of thoracic pain in a group of nulliparous young adult women in order to begin exploring the questions: Do larger breasted women experience more thoracic pain than small breasted women? Could an incorrectly fitted or sized bra contribute to women's thoracic pain? Clarification of these relationships may aid in the care of women presenting with thoracic pain.

## Method

This study was approved by the Victoria University Human Research Ethics Committee. All participants provided written informed consent for their participation in the study.

### Participants

Thirty women (18–26 years) with self-reported posterior thoracic pain, who wore bras during daytime hours, volunteered to participate in the study. Posterior thoracic pain was defined as pain felt anywhere in the posterior aspect of the thoracic cage, in the region bordered by first ribs and first thoracic vertebra superiorly and the twelfth vertebra and ribs inferiorly, and including the periscapular areas.

Recruitment posters for this study, displayed at Victoria University (City Flinders campus), invited women aged between 18 and 50 years, who regularly wore bras (not strapless) during the daytime but not during nighttime sleeping, and were currently experiencing non-specific "upper back pain" to volunteer for this study. Volunteers were excluded if at the time of the study, or in the three months prior or one month following, they: (a) were able to report specific pathology that explained their posterior thoracic pain, (b) were pregnant, breast-feeding, or expressing breast milk, (c) were menopausal or experiencing symptoms possibly attributable to menopause, (d) reported breast changes related to commencing or ceasing use of an oral contraceptive pill, or (e) reported body weight gain or loss of more than 5 kg.

### Measures

Data collection for this study comprised 4 steps. Participants completed a screening survey to ensure inclusion criteria were met and to estimate menstrual cycle stage (see Additional file 1), and a self-report measure of pain nature and intensity (short-form McGill Pain Questionnaire [17]). Current bra fit was assessed using observation criteria for bra fit (Triumph International; see Additional file 2). Actual bra size, as an estimate of breast size, was assessed by band and cup size measurements using established international guidelines [15].

Menstrual cycle stage: The typical 28 day menstrual cycle was divided into 4 stages of approximately one week each. Numerical labels from 1 to 4 were attributed to menstrual cycle stages (1 = pre-menstrual, 2 = menstruating, 3 = post-menstrual, 4 = mid-cycle). Participants were asked to self-report their menstrual cycle stage by recalling the date of their most recent menstruation and counting forward in weeks.

Short-form McGill Pain Questionnaire [17]: Three numerical scores of pain were derived from sub-scales (total pain, sensory pain, affective pain) of the short-form McGill Pain Questionnaire. Two further sub-scales, the present pain index (PPI) and a visual analogue scale (VAS), returned categorical (range 0–5, 0 = no pain, 5 = excruciating) and numerical measures (range 0–100, 0 = no pain, 100 = worst possible pain) of the severity of current pain.

#### Bra Fit

Categories of bra fit were allocated numerical scores in the range -4 to 4, with 0 indicating a correctly fitted bra. 4 observational criteria, each scored +1 if present, were used to determine if a bra was too large, and another 4 criteria, each scored -1 if present, were used to determine if a bra was too small. The overall score of bra fit was the sum of scores for each criterion. A negative score indicated that the current bra worn was too small and a positive score indicated that the current bra was too large. The numerical part of score indicated the net number of criteria on which a bra was identified as poorly fitted.

#### Bra size

**Figure 1:**
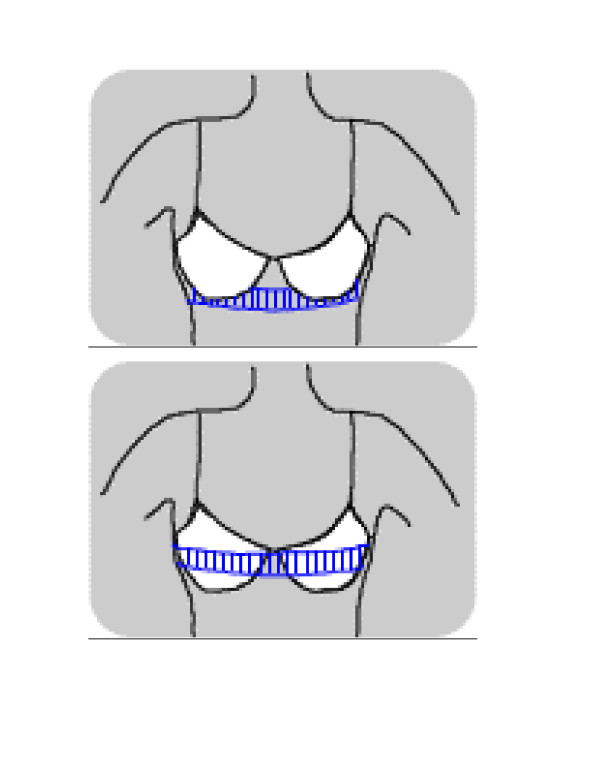
Bra size measurements.

Bra size measures yielded two numerical scores: a) actual bra size [15], which was assumed to be an estimate measure of breast size, and b) difference between bra size worn and that measured as actual bra size (bra size difference). Bra size was a two part measure comprising cup size and band size (see Figure 1). Cup size is thoracic circumference across the fullest part of the breasts, converted to categorical classification ranging from AA (smallest) to F (largest in this study). Band size is thoracic circumference under the bust at the level of the inframammary fold, converted to categorical classification ranging from 10 to 22, approximately equal to dress size [13]. Bra size difference score was also a two part score, comprising a sign that indicated whether the bra worn was too small (negative) or too large (positive), and a numerical score that indicated the number of bra size categories between the bra worn and the bra fitted.

### Data Analysis and Conventions for Interpretation

Pearson's correlation co-efficients (r) were calculated to determine the strength and direction of linear relationships between pairs of numerical variables. Effect sizes were calculated as r^2^. Consistent with Cohen's conventions, correlations were interpreted according to size as well as direction [18]. Correlations of less than 0.3 are described as small or weak, between 0.3 and 0.5 are medium or moderate, and greater than 0.5 are large or strong [18,19].

## Results

Thirty young women (18–26 years) participated in this study, and 26 women provided complete data sets. Summary data for each participant are provided in Table 1.

**Table 1:** Summary of data set for each participant

**Participant**	**Age**	**Pain duration**	**Bra fitted**	**Bra Size (score)**	**Bra Fit (score)**	**Bra size worn**	**Bra Size Difference**	**Menstrual Stage**	**Total Pain**	**Sensory Pain**	**Affective Pain**
1	18	5	12A	4	0	10A	-1	post	16	12	4
2	18	4	10B	4	2	10B	0	pre	3	3	0
3	18	3	10B	4	-1	10C	1	men	15	11	4
4	26	4	12D	7	-1	12C	-1	men	6	5	1
5	18	6	10D	6	0	10D	0	-	4	3	1
6	18	6	10C	5	-1	10B	-1	pre	6	5	1
7	18	6	16DD	10	-3	14C	-3	mid	10	8	2
8	18	6	12DD	8	-1	12D	-1	post	5	4	1
9	18	6	10C	5	1	10B	-1	pre	6	5	1
10	18	6	12D	7	-2	12C	-1	mid	5	5	0
11	18	6	10C	5	-1	12B	0	post	5	5	0
12	18	6	14E	10	-1	12DD	-2	post	1	1	0
13	18	6	14F	11	-1	14D	-3	mid	7	7	0
14	18	6	10D	6	-2	10C	-1	pre	15	11	4
15	18	6	10E	8	-3	10D	-2	pre	2	2	0
16	18	6	12AA	3	1	12B	2	mid	7	6	1
17	18	5	12DD	8	-2	12D	-1	mid	7	5	2
18	18	5	12DD	8	1	14B	-2	-	10	9	1
19	18	5	10D	6	0	10B	-2	post	11	11	0
20	18	6	12D	7	-1	12C	-1	mid	9	8	1
21	18	2	10C	5	0	12B	0	pre	4	4	0
22	18	6	12B	5	0	10B	-1	-	5	5	0
23	18	3	10B	4	2	12B	1	-	5	5	0
24	19	4	12D	7	-3	12B	-2	post	9	7	2
25	18	6	10A	3	-1	10AA	-1	am	12	12	0
26	18	5	10D	6	-1	10C	-1	men	5	5	0
27	18	6	10D	6	-3	10C	-1	men	6	4	2
28	18	3	12DD	8	-3	12C	-2	mid	5	5	0
29	18	5	12A	4	0	12A	0	pre	7	7	0
30	18	6	14B	6	2	10D	0	pre	13	7	6

Pain duration: Categorical scores 1 to 6 indicate self-reported duration of pain. 1 = <2 week, 2 = <1 month, 3 = <3 months, 4 = <6 months, 5 = 6–12 months, 6 = >1 year

Menstrual stage: Categorical labels indicate self-reported stage of menstrual cycle. Pre = week before menstrual period, men = currently menstruating, post = week following menstrual period, mid = mid-cycle, approximately 2 weeks following last menstrual period, am = amenorrhea, no regular menstrual cycle.

### Missing data

Four women did not respond to the survey item regarding current stage of their menstrual cycle, and one participant reported amenorrhea, so these participants' data were excluded from some analyses. Two women omitted the VAS and PPI of the McGill pain questionnaire. Four women returned zero scores (no pain) on the VAS and seven women returned zero scores (no pain) on the PPI, but each of these women reported some current pain on the word lists of the McGill pain questionnaire. Because of these discrepancies in the data set, the VAS and PPI sub-scales were excluded from the analyses.

**Figure 2:**
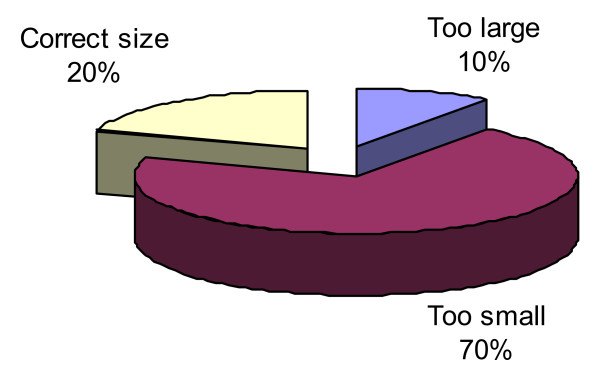
Categorical Classification of Bra Fit.

Bra fit scores revealed that the majority (80%) of participants were wearing bras that were was the wrong size for them, with 70% wearing bras that were too small and 10% wearing bras that were too large (see Figure 2). Bra size difference scores ranged from 1 to -3, with a clustering of scores at the negative end of this range, indicating that most women self-selected bras that were too small (see Table 1).

A large negative correlation (r = -0.78) was identified between actual bra size (breast size) and bra size difference. These results indicate that there was a strong linear relationship between the size of women's breasts and the size of bras they selected for themselves. It appears that women do not simply choose bras in the wrong size. More specifically, the larger a woman's breasts, the more likely she will be wearing bras that were too small, and conversely, the smaller a woman's breasts, the more likely she will choose bras that were too big.

Bra size difference and bra fit were strongly positively correlated (r = 0.55). This relationship was unsurprising, indicating that the worse a bra fitted the more likely that bra was the wrong size for the woman wearing it. A moderate negative correlation was also found between breast size and bra fit (r = -0.50), suggesting that larger breasted women were more likely to be wearing ill fitting bras.

A small negative correlation was seen between breast size and total pain (r = -0.23). Negligible correlations were identified between bra fit and self-reported scores of both total pain (r = 0.036) and sensory pain (r = 0.032).

Moderate correlations were found between menstrual cycle stage and both bra fit (r = 0.32) and bra size difference (r = 0.29).

## Discussion

That 80% of participants were wearing incorrectly sized bras is consistent with previous studies [11,20]. Bra-sizing and fitting are learned skills and may be difficult to perform on oneself. Most women are not trained in bra-sizing, but make bra purchasing decisions unassisted. Annual professional bra-sizings are recommended, but many women do not seek these [11], possibly because bra-sizing services are typically undertaken by bra salespeople, leaving women feeling somewhat compelled to purchase bras from the people conducting the sizings. Also, women, particularly larger busted women, may experience feelings of embarrassment and self-consciousness during bra sizings, tempting women to avoid such appointments and attempt to size and fit their own bras.

Interpreting the large negative correlation between breast size and bra size in conjunction with the moderate negative correlation between breast size and bra fit and our participants' tendency to self-select bras that are too small, we suggest that women with large breasts are more likely than their small breasted counterparts to be wearing incorrectly sized and fitted bras. There are several possibly explanations for why being large breasted is particularly associated with wearing a bra that is poorly fitted or the wrong size. Measurement of the underband and overbust is a reasonably accurate for cup sizes ranging between AA and C, but declines somewhat for the larger cup sizes (D through to F) [20]. Put simply, it is easier to accurately size bras for smaller breasted women. Larger breasted women are more prone to incorrect bra sizing because their breasts may be ptotic and bulbous, making accurate overbust measurement difficult. When taking the underband measurement there is a tendency to cut into excess flesh with the tape measure magnifying the inaccuracy of cup-size measurement [11]. Accurate bra fit is similarly difficult for overweight and obese women [13]. When discussing this study at a conference, a "well-endowed" colleague suggested that we plan a follow up study recruiting women with breast sizes DD and larger in order to further explore these relationships among larger breasted women in particular.

That the negative correlation between breast size and bra fit was only moderate, rather than large like the correlation between breast size and bra size, suggests that women may be able to somewhat compensate for selecting incorrect bra sizes through the fitting adjustments built into most bras. In the underwear industry, there is some understanding of bra size equivalence; for example, a 10C bra can be adjusted to fit a 12B woman by shortening the shoulder straps and lengthening the underbust band [15]. We have taken this equivalence into consideration when assessing bra fit, and this overlap in fit between sizes also contributes to explaining why these two negative correlations are not of approximately equal magnitude.

Although bra fit appears unrelated to pain, if a bra is poorly fitted, bra function (eg: breast support, reduction of breast bounce) may be compromised. Bras are potentially expensive, rarely seen, underwear items. It is likely that many women do not replace their bras regularly. Like all items of clothing, the shape and structure of bras may deteriorate with age, use, and laundering, and a bra that fitted well at the time of purchase, might not fit so well months or years later.

In this study, small breast size correlated somewhat with greater severity of self-reported pain. Although this correlation was small, it appears to contradict the results of most studies of reduction mammaplasty in which participants reported either complete or partial reduction in thoracic pain following surgical reduction of breast mass [3,6-10,21,22]. The effect size corresponding to a correlation of -0.23 is r^2 ^= 0.04, indicating that only 4% of the variance is pain scores is accounted for by breast size. Using the McGill Pain Questionnaires (including short-form) participants are required to report pain severity according to various descriptors of pain (eg: burning, nagging, crushing). Because these instruments probe an individual's perception of pain, they may not be ideal instruments for comparing pain severity between individuals. Pain is a personal phenomenon, and 4% variance in pain across a group may be associated, genuinely or otherwise, with almost anything [17].

Our results do not explain pain as a correlate of breast size. Note that most of the current evidence that large breasts explain pain is based on post-surgical data [3,6-10,21,22] and recent case review suggests that reduction in breast mass might not be the variable of primary importance [2]. In 1993 Gonzalez et al [4] re-defined macromastia, removing specific reference to breast size and emphasizing clinical symptoms and functioning. We concur with Gonzalez et al's view that symptoms and function are likely to be more important than breast size per se. Although we acknowledge that women may have multiple reasons for seeking breast reduction surgery, we suggest that pain is likely to be a primary motivator, and that the sample of women volunteering for post-surgical studies might not be representative of macromastic women.

Participants in our study were young women students, and none had ever been pregnant. Other possible correlates of thoracic spinal pain, such as prolonged study postures and emotional stress, need to be considered in these participants. In young non-pregnant women, thoracic pain is probably multifactorial rather than directly related to breast size or bra fit. Participants in previous studies linking breast size to thoracic pain were aged from 32 to 40.6 years [3,6-10,21,22] and some participants had born and breast fed children. Breast morphology is likely to differ between these groups.

Female breasts are affected by hormonal changes associated with menstruation, pregnancy, menopause, and some pathology [4,5]. Monthly fluctuations of estrogen and progesterone are believed responsible for the common changes, including increased breast size and tenderness that many women experience in the week preceding their period [23]. In our sample, menstrual cycle stage correlated moderately with both bra fit and bra size difference, suggesting that women may require bras of a different fit or size at different stages of their menstrual cycles. These results have implications for future research and also for the underwear industry [24]. We recommend that stage of menstrual cycle be accounted for as a confounding factor in future research designs.

In designing this study, it was not possible to account for all possible correlates of thoracic pain among our young women. We acknowledge that participants' occupations, sporting activities, and other daily habits may have confounded our results. Also, we did not take any anthropometric measures in this study, nor correlate such variables with breast size, bra fit, or back pain. We acknowledge that body mass, and in particular, percentage body fat, may influence breast size and possibly breast mass [4,5,13,20]. We reiterate that the purpose of this study was to explore the relationship between bra fit, breast size, and thoracic pain. If a strong and consistent relationship between breast size and thoracic pain were identified, then future research and clinical interventions might reasonably be directed towards investigation of variables possibly correlated with breast size.

Small sample size and limited age range compromises the generalisability of this research. Follow-up studies are needed to establish a more comprehensive information base and we recommend that these studies include more women across the range of adulthood, from various occupational groups, and with diverse levels of current and past physical activity engagement. We recommend that future research in this area further explores correlates of macromastia and thoracic spinal pain, and investigates alternative treatment methods to reduction mammaplasty for relieving macromastic pain.

## Conclusion

This point in time snapshot of young adult women students reporting thoracic spinal pain suggests that there is little meaningful correlation between breast size and pain intensity, or between pain and bra fit. Breast size correlated strongly and negatively with bra size, and moderately with bra fit, but was not highly correlated with pain severity.

## Competing interests

The author(s) declare that they have no competing interests.

## Authors' contributions

This study was completed by KW in partial fulfillment of a Masters degree. MC and KF acted as supervisors. KW conceived the idea for the study. KW and MC designed the study and sought ethical approval. KW collected the data. KW and MC analysed the data. KF assisted in supervision when MC moved to another department partway through the study. All authors contributed to writing this manuscript, and reviewed and edited this manuscript for publication.

## Supplementary Material

Additional file 1 Screening survey. Screening survey administered to collect demographic data and ensure that all participants satisfied inclusion criteria. Click here for file

Additional file 2 Observational criteria for bra fit. Checklist used to determine whether the bra worn was too large or too small, and to what extent the fit differed from Triumph International guidelines. Click here for file
