# Supplementary material for: Breast size, bra fit and thoracic pain in young women: a correlational study
Source: Chiropr Osteopat. 2008 Mar 13;16:1. doi: 10.1186/1746-1340-16-1 (PMC2275741; doi:10.1186/1746-1340-16-1)
Supplement: Additional file 1 — Screening survey. Screening survey administered to collect demographic data and ensure that all participants satisfied inclusion criteria. [file 1746-1340-16-1-S1.doc]

**Screening survey**

Unless indicated please tick the appropriate box.

1. Which age group is applicable to you?

□18-25years □ 26-35years □36-50years □ > 50years

1. What bra size do you most commonly wear?

10 AA A B C D DD E F

12 AA A B C D DD E F

14 AA A B C D DD E F

16 AA A B C D DD E F

Other

1. Has your bra sized changes within the past 6months? □ Yes □ No

If yes, what was your previous bra size? Please state

1. Do you suffer from thoracic and/or posterior chest wall pain? □ Yes □ No

If yes, how long have you been experiencing this pain?

□<2weeks □ <1month □ <3months □ <6months □ 6-12months □ >1year

1. Do you use the oral contraceptive pill (OCP)? □ Yes □ No

If yes, have you changed OCP within the past 6months? □ Yes □ No

If yes, have you experienced any breast related changes? □ Yes □ No

1. Regarding menstruation, are your periods regular? □ Yes □ No
2. Do you experience any changes in breast size during your menstrual cycle?

□ Yes □ No

If yes, at what stage of your cycle do these changes occur?

1. Currently, at what stage of your menstrual cycle are you?
2. Is your weight steady and constant? □ Yes □ No
3. Have you experienced any recent (within 3months) weight gain/loss?

□ Yes □ No

If yes, what is the weight difference?

11. Have you ever been pregnant? □ Yes □ No

If yes, how many pregnancies have you had?

If yes, when was your last pregnancy?

12. Have you in the past or are you currently experiencing menopausal symptoms?

□ Yes □ No
