# Supplementary material for: Breast size, bra fit and thoracic pain in young women: a correlational study
Source: Chiropr Osteopat. 2008 Mar 13;16:1. doi: 10.1186/1746-1340-16-1 (PMC2275741; doi:10.1186/1746-1340-16-1)
Supplement: Additional file 2 — Observational criteria for bra fit. Checklist used to determine whether the bra worn was too large or too small, and to what extent the fit differed from Triumph International guidelines. [file 1746-1340-16-1-S2.doc]

Observational criteria for bra fit

Participant Number:

Underbust Measurement: Overbust Measurement: Bra Size:

Observation criteria for bra if too small:

1) Bust bulging over the top? Yes No

2) Centre of front sitting away from body? Yes No

3) Bust bulging at the underarm? Yes No

4) Back of the bra cutting into the back and cutting bulging? Yes No

Observation criteria for bra if too large:

1) Is the cup wrinkled? Yes No

2) Shoulder straps slipping down the shoulder? Yes No

3) Back of bra riding up towards the neck? Yes No

4) Cups of bra sitting high at the underarm? Yes No
